# The Influence of Emotion on Keyboard Typing: An Experimental Study Using Auditory Stimuli

**DOI:** 10.1371/journal.pone.0129056

**Published:** 2015-06-11

**Authors:** Po-Ming Lee, Wei-Hsuan Tsui, Tzu-Chien Hsiao

**Affiliations:** 1 Institute of Computer Science and Engineering, National Chiao Tung University, Hsinchu, Taiwan, R.O.C; 2 Institute of Biomedical Engineering, National Chiao Tung University, Hsinchu, Taiwan, R.O.C; 3 Department of Computer Science, National Chiao Tung University, Hsinchu, Taiwan, R.O.C; 4 Biomedical Electronics Translational Research Center and Biomimetic Systems Research Center, National Chiao Tung University, Hsinchu, Taiwan, R.O.C; Ulm University, GERMANY

## Abstract

In recent years, a novel approach for emotion recognition has been reported, which is by keystroke dynamics. The advantages of using this approach are that the data used is rather non-intrusive and easy to obtain. However, there were only limited investigations about the phenomenon itself in previous studies. Hence, this study aimed to examine the source of variance in keyboard typing patterns caused by emotions. A controlled experiment to collect subjects’ keystroke data in different emotional states induced by International Affective Digitized Sounds (IADS) was conducted. Two-way Valence (3) x Arousal (3) ANOVAs was used to examine the collected dataset. The results of the experiment indicate that the effect of arousal is significant in keystroke duration (p < .05), keystroke latency (p < .01), but not in the accuracy rate of keyboard typing. The size of the emotional effect is small, compared to the individual variability. Our findings support the conclusion that the keystroke duration and latency are influenced by arousal. The finding about the size of the effect suggests that the accuracy rate of emotion recognition technology could be further improved if personalized models are utilized. Notably, the experiment was conducted using standard instruments and hence is expected to be highly reproducible.

## Introduction

Graphics and the computing capabilities of computers have become powerful recently. However, a computer interactive application that does not understand or adapt to a users’ context could still lead to usability problems. The users’ context mentioned here is used as a general term to cover factors related to users, that may include the condition of a user, the goal that the user intend to achieve, and the users’ preference of the system response. An application that is not aware of the context of its users could provide annoying feedback, interrupt users in an inappropriate situation, or increase the users’ frustration [1]. In 1990s, Rosalind W. Picard, the mother of “Affective Computing”, began to propose and demonstrate her ideas about having computers identify a user’s emotional state and about the related possible improvements to the computer applications [2]. Subsequently, many approaches for detecting users’ emotions have been demonstrated to be useful. For instance, emotion recognition by facial expression, which aims to model visually distinguishable facial movements [3]; by speech, for which researchers utilize acoustic features such as pitch, intensity, duration, and spectral data [4]; and by physiological data, such as the heart rate and sweat [5]. In the past two decades, substantial amount of research with regard to affective computing has been conducted in the field of Human-Computer Interaction (HCI) [1, 6–22], and has also been recognized by the application field (e.g., in the tutoring system research [23–35]).

Emotion recognition technology based on keystroke dynamics was not reported in the literature until Zimmermann, Guttormsen (36] first described this approach. The authors proposed an experiment designed to examine the effect of film-induced emotional states (PVHA, PVLA, NVHA, NVLA and nVnA (P = positive, N = negative, H = high, L = low, n = neutral, V = valence, A = arousal) in subjects, with the keystroke dynamics in regard to keystroke rate per second, average duration of keystroke (from key-down until key-up event). However, they did not actually carry out the work described in their proposal. The use of keystroke dynamics for emotion recognition has two main advantages that make such a technique favorable. The two advantages are that it is non-intrusive and easy-to-obtain because the technique does not require any additional equipment or sensors other than a standard input device, which is the keyboard of a computer. Since 2009, numerous studies in the field of computer science have reported the development of emotion recognition technology based on keystroke dynamics. Vizer, Zhou (37] reported the use of ratios between specific keys and all keys to recognize task-induced cognitive and physical stresses from a neutral state. The authors achieved a classification rate of 62.5% for physical stress and 75% for cognitive stress. The key ratios could represent the frequencies of typing specific keys, which may increase or decrease due to the changes in emotional state. The analysis result was produced based on sophisticated Machine-Learning (ML) algorithms, and hence, the relationship between emotion and these ratios was not identified. Notably, most of the main streams of the ML algorithms only produce models that are considered to be a black box, and do not produce models that is described clearly, with the relationship between independent variable and dependent variable identified and could be easily interpreted. The ML algorithms are usually used for building models from dataset that contains complex relationships which are not able to be identified by a traditional statistical model (e.g., t-test, ANOVA). In 2011, Epp, Lippold (1] reported a result of building models to recognize experience-sampled emotional states based on the keystroke durations and latencies that were extracted from a fixed typing sequence. The accuracy rates of classifying anger, boredom, confidence, distraction, excitement, focus, frustration, happiness, hesitance, nervousness, overwhelmed, relaxation, sadness, stress, and tired, with respect to two-class models that classify instances into two classes (i.e. is an instance with the target label or not with the target label), were 75% on average. The latency features can be understood as the speed of typing on the keys [38], whereas the duration features may be understood as the force used for pressing the keys [21]. The study [1] built the model by using the ML algorithms and also a correlation-based feature subset attribute selection method [39]. Although the keystroke features that were used to build the model with the highest accuracy rate were reported, the relationship between emotion and keystroke dynamics, still, was not provided. Notably, latest study in the area of psychophysiology [21] examined Heart Rate (HR), Skin Conductance Response (SCR), and the dynamics of button presses after an unexpected delayed system response of a user interface. The study [21] reported that the immediate feedback trials that followed delayed feedback trials showed a significant higher SCR, lower HR, stronger button press intensity, and longer duration compared to trials that followed immediate feedback trials. Furthermore, more results related to classification on emotional data using feature set similar to the feature sets used in the previous studies [1, 36, 37] have been proposed recently. Alhothali (40] reported the use of keystroke features that were extracted from arbitrarily typed keystroke sequences as reaching an 80% accuracy rate of classifying experience-sampled positive and negative emotional states. Bixler and D'Mello (41] demonstrated a 66.5% accuracy rate on average for two-class models in detecting boredom, engagement, and neutral states, for which the emotional data used were collected using the experience sampling method.

By applying ML methodology for building classification models from various datasets collected from different experimental setups, these studies have suggested that keystroke duration and latency can be used for model building. One therefore could hypothesize that the keystroke duration and latency may be different when subjects are in different emotional states. However, the details about the relationship between keystroke dynamics and emotions were never discussed in previous studies [1, 37, 40, 41] possibly due to the limitation of the adopted methodology. Specifically, the methodology used does not allow previous studies to come up with clear hypotheses due to a lack of specificity with regard to the exact parameters that were used to classify the data. This makes the studies [1, 37, 40, 41] examples and showcases. The current study aimed to test the hypotheses that keystroke dynamics may be influenced by emotions. We argued that the relationship between keystroke dynamics and emotion should not be too complex. Based on a rigorous experimental setup, traditional statistical methods could be used to examine the variance and reveal the relationship, without the use of sophisticated ML algorithms. The current study examined the variance of keystroke dynamics caused by emotions. Specifically, three hypotheses were tested. It was hypothesized that difference in keystroke dynamics due to different emotional states would appear in keystroke duration, keystroke latency, and the accuracy rate of a keyboard typing task. This study aimed to answer two research questions. First, do the variance in the keystroke features that are ordinarily used for model building (i.e. keystroke duration, keystroke latency, accuracy rate) in previous studies exceeds significance level under different emotional states? Second, how large are the variances contributed by emotions in these keystroke features? Furthermore, as suggested in earlier studies [21, 38], we expected a significantly longer keystroke duration to negative emotional stimuli.

## Materials and Method

### Ethics Statement

This study was under the research project “A study of interactions between cognition, emotion and physiology (Protocol No: 100-014-E),” which was approved by the Institution Review Board (IRB) of the National Taiwan University Hospital Hsinchu Branch. Written Informed consents were obtained from all subjects before the experiment.

### Subjects

Fifty-two subjects ranging in age between 20 and 26 (M = 21.3, SD = 1.2; 44 men, 8 women) performed keyboard typing tasks right after presented with emotional stimuli. The subjects were college students selected from a university in Taiwan, with normal hearing in regard to relative sensitivity at different frequencies. All the subjects self-reported that they were nonsmoker, healthy, with no history of brain injury and cardiovascular problems. The subjects also reported that they had normal or corrected-to-normal vision and normal range of finger movement. They are all right-handed.

### Experimental Procedure

A subject wore earphones during the experiment and was instructed to type-in a target typing text "748596132" once immediately after hearing each of the International Affective Digitized Sounds 2nd edition (IADS-2) [42] sounds, for 63 trials. The experiment was conducted based on a simple dimensional view of emotion, which assumes that emotion can be defined by a coincidence of values on two different strategic dimensions that are, valence and arousal. To assess these two dimensions of the affective space, the Self-Assessment Manikin (SAM), an affective rating system devised by Lang [43] was used to acquire the affective ratings.

**Fig 1:**
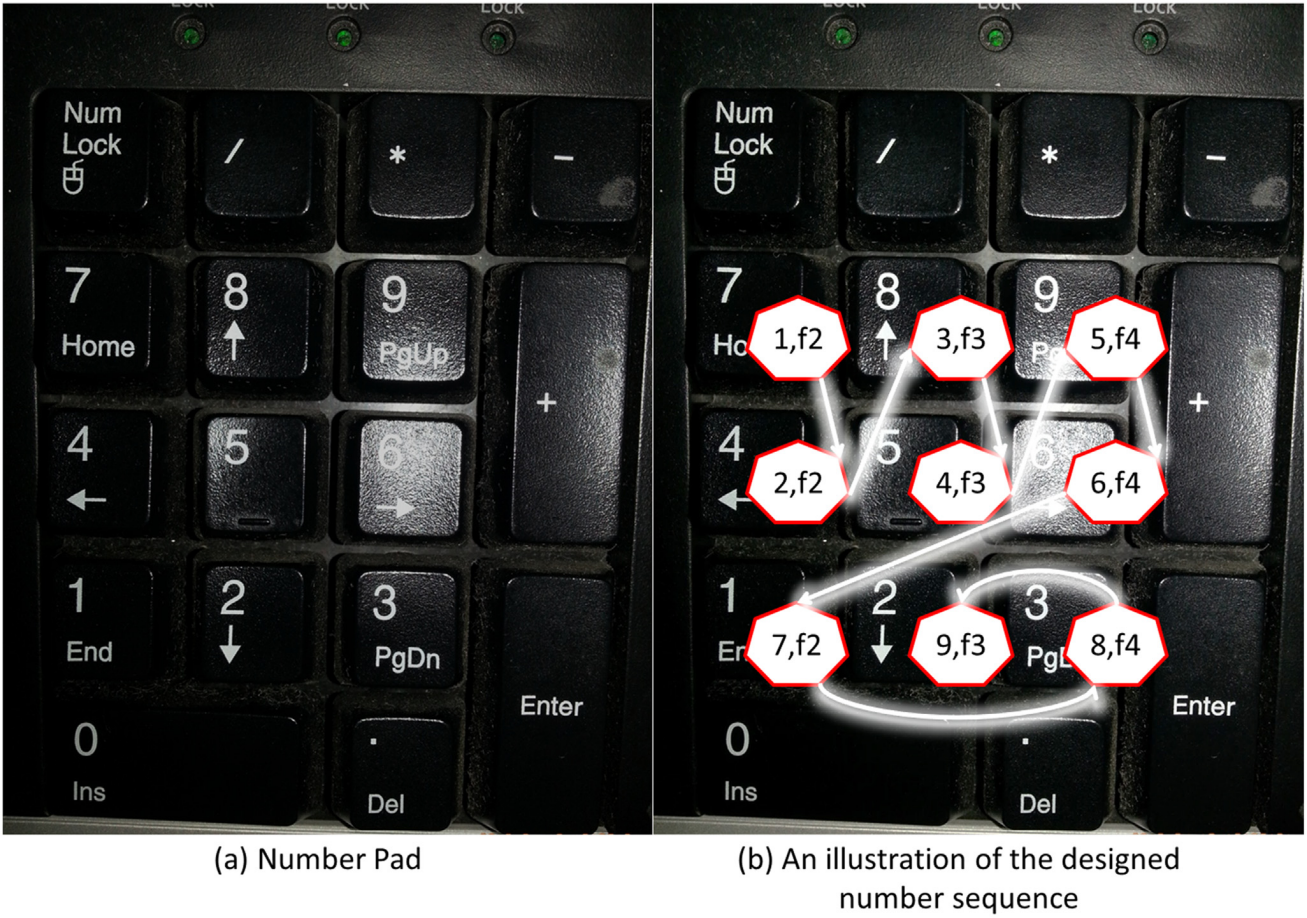
The number pad in the keyboard used in our experiment, with an illustration of the design concept of our designed target number typing sequence. The arrow shows the order of changes of the typing target. For those (*x*, *y*) pairs in the heptagons, *x* represents the order of a typing target and *y* represents the desirable finger (i.e. thumb (f1), index finger (f2), middle finger (f3), ring finger (f4), and little finger (f5) or pinky) that was used for typing the corresponding typing target.

Each trial began with an instruction (“Please type-in the target typing text after listening to the next sound”) presented for 5 s. Then, the sound stimulus was presented for 6 s. After the sound terminated, the SAM with a rating instruction (“Please rate your feeling on both the two dimensions after typing the target typing text ‘748596132’”) was presented. The subject first typed-in the target typing text once, and then made his/her ratings of valence and arousal. A standard 15 s rating period was used, which allows ample time for the subject to make the SAM ratings. A computer program controlled the presentation and timing of the instructions and sounds. The keystroke data was recorded during the typing task. In addition to the 63 trials, 3 practice trials and a training section were applied prior to the experiment. Three sounds (birds, female sigh, and baby cry) provided the subject with a rough range of the types of the contents that were presented. After these practice trials was the training section, in which the subject continually typed-in the target typing text (presented on the screen by blue text and gray background) using the number pad (shown in Fig 1(a)) that is located on the right side of a standard keyboard, for 40 s.

A number sequence was used as the target typing text instead of an alphabet sequence or symbols to avoid possible interference caused by linguistic context to the subject’s emotional states. In all the various number sequences used in our pilot experiments [38, 44], we found the existence of the difference in keystroke typing between the subjects in different emotional states. However, we also found that the relationship between the keystroke typing and emotional states may be different due to different keys that are typed and also the order of typing. A comparison of keystroke typing between emotional states using different number sequences may reduce the power of statistical tests (given a same number of trials). Hence, to conduct a more conservative comparison across emotion and to enhance the generalizability of this study, we decided to use a single number sequence that is designed to be general. We designed the target typing text “748596132” to 1) be easy to type without requiring the subjects to perform abrupt changes in their posture, 2) have the number of digits fairly distributed on a number pad, and 3) encourage all the subjects to maintain a same posture (i.e., in terms of finger usage) when typing the given sequence [38] (see Fig 1(b) for more detail). The time length of the experiment was designed to be as short as possible to avoid the subjects from being tired of typing on the keyboard. Note that all the subjects indeed reported that they were not fatigued after the experiment.

### Stimuli and Self-Report

The stimuli we used were 63 sounds selected from the IADS-2 database, which is developed and distributed by the NIMH Center for Emotion and Attention (CSEA) at the University of Florida [42]. The IADS-2 is developed to provide a set of normative emotional stimuli for experimental investigations of emotion and attention and can be easily obtained through e-mail application. The IADS-2 database contains various affective sounds proved to be capable of inducing diverse emotions in the affective space [45]. The sounds we used as the stimuli were selected from IADS-2 database complying the IADS-2 sound set selection protocol described in [42]. The protocol includes the constraint about the number of sounds used in a single experiment, and the distribution of the emotions that are expected to be induced by the selected sounds. Two different stimulus orders were used to balance the position of a particular stimulus within the series across the subjects. The physical properties of these sounds were also controlled to prevent clipping, and to control for loudness [42].

The SAM is a non-verbal pictorial assessment designed to assess the emotional dimensions (i.e. valence and arousal) directly by means of two sets of graphical manikins. The SAM has been extensively tested in conjunction with the IADS-2 and has been used in diverse theoretical studies and applications [46–48]. The SAM takes a very short time to complete (5 to 10 seconds). For using the SAM, there is little chance of confusion with terms as in verbal assessments. The SAM was also reported to be capable of indexing cross-cultural results [49] and the results obtained using Semantic Differential scale (the verbal scale provided in [50]). The SAM that we used was identical to the 9-point rating scale version of SAM that was used in [42], in which the SAM ranges from a smiling, happy figure to a frowning, unhappy figure when representing the affective valence dimension. On the other hand, for the arousal dimension, the SAM ranges from an excited, wide-eyed figure to a relaxed, sleepy figure. The SAM ratings in the current study were scored such that 9 represented a high rating on each dimension (i.e. positive valence, high arousal), and 1 represented a low rating on each dimension (i.e. negative valence, low arousal).

### Apparatus

During the experiment, a subject wore earphones (Sennheiser PC160SK Stereo Headset) and sat on an office chair (0.50 x 0.51 m, height 0.43 m), in a small, quiet office (7.6 x 3.2 m) without people. The office was with window and the ventilation was guaranteed. The computer system (acer Veriton M2610, processor: Intel Core i3-2120 3.3G/3M/65W, memory: 4GB DDR3-1066, operating system: Microsoft Windows 7 Professional 64bit) used by the subject was put under a desk (0.70 x 1.26 m, height 0.73 m). The subject was seated approximately 0.66 m from the computer screen (ViewSonic VE700, 17 inch, 1280 x 1024 in resolution). The keyboard used by the subject was an acer KU-0355 (18.2 x 45.6 cm, normal keyboard with the United States layout, typically used for Windows operating system) connected to the computer system used through USB 2.0 communication interface. The distance between the center of adjacent keys (size: 1.2 x 1.2 cm) of the number pad used was 2 cm. Keyboard lifts (the two small supports at the back of the keyboard) which raise the back of the keyboard for 0.8 cm when used, were not used in this experiment. The subject was sat approximately 0.52 m from the center of the number pad (i.e. the digit “5” of the number pad). The keystroke collection software was developed using C# project built by using Visual Studio 2008 and was executed on the. NET framework (version 3.5) platform. The reason of using C# programming language in developing this software was that the language provides more sufficient Application Programming Interfaces (APIs) for utilizing the function of keystroke-interrupt detection in Microsoft Windows operation systems than other programming languages such as R, Matlab, Java, and Python.

### Data Analysis

In total, 63 (trials) x 52 (subjects) = 3,276 rows of the raw data were collected during the experiment. However, 117 (3.6% of the 3276 samples) rows of the raw data were excluded because the SAM rating was not completed. In our analysis, a sequence typed is a "correctly typed sequence" if the target typing text was correctly typed and “incorrectly typed sequence” if incorrectly typed. For instance, if a subject typed “7485961342”, of which the “4” at the 9^th^ digit is misplaced, the sequence typed was considered as an incorrectly typed sequence. A pre-processing routine was applied to the raw data to separate all the correctly typed sequences from incorrectly typed sequences. Keystroke duration and keystroke latency features were only extracted from the correctly typed sequences (91.2% of the 3,024 samples). The keystroke duration is the time that elapsed from the key press to the key release, whereas the keystroke latency is the time that elapsed from one key release to the next key press [51].

The extracted keystroke duration and keystroke latency features were submitted to two two-way 3 (Valence: negative, neutral, and positive) x 3 (Arousal: low, medium, and high) Repeat Measures ANOVAs [52], respectively. To analyze the accuracy rate of keyboard typing, the accuracy data (0 for incorrectly typed sequence and 1 for correctly typed sequence) of all the typed sequences was submitted to a two-way 3 (Valence: negative, neutral, and positive) x 3 (Arousal: low, medium, and high) Repeat Measures ANOVA. Post-hoc analysis was conducted using multiple t-tests with Bonferroni correction.

The 9-point scale SAM ratings of the valence and arousal were translated into three levels of the ANOVA factor Valence and Arousal. Eleven subjects were excluded from the application of the Repeat Measures ANOVA (leaving 2,583 rows of the raw data) because of having numerous empty cells. These subjects reported a small range of changes in SAM ratings (i.e. unsuccessful emotion elicitation) throughout the experiment, which leaded to empty cells. Specifically, we removed these 11 subjects that contain over 3 empty cells (missing values) in a 3 (Valence: negative, neutral, and positive) x 3 (Arousal: low, medium, and high) table. The decision of not to impute them was because of that the research objectives of the current study were to examine the keystroke dynamics in the 3 x 3 emotional conditions, of which the multiple imputations may lead to unreliable results. Notably, the 11 subjects that were removed from the analysis contain 6, 6, 6, 5, 4, 4, 4, 3, 3, 3, and 3 empty cells. The ANOVA results of the dataset that included these subjects by imputing all the missing values by using average values are also presented in the result section, next to the ANOVA result of the dataset that with these subjects excluded. The significance level α of the entire statistical hypothesis tests used in this paper was set to 0.05.

## Results

**Fig 2:**
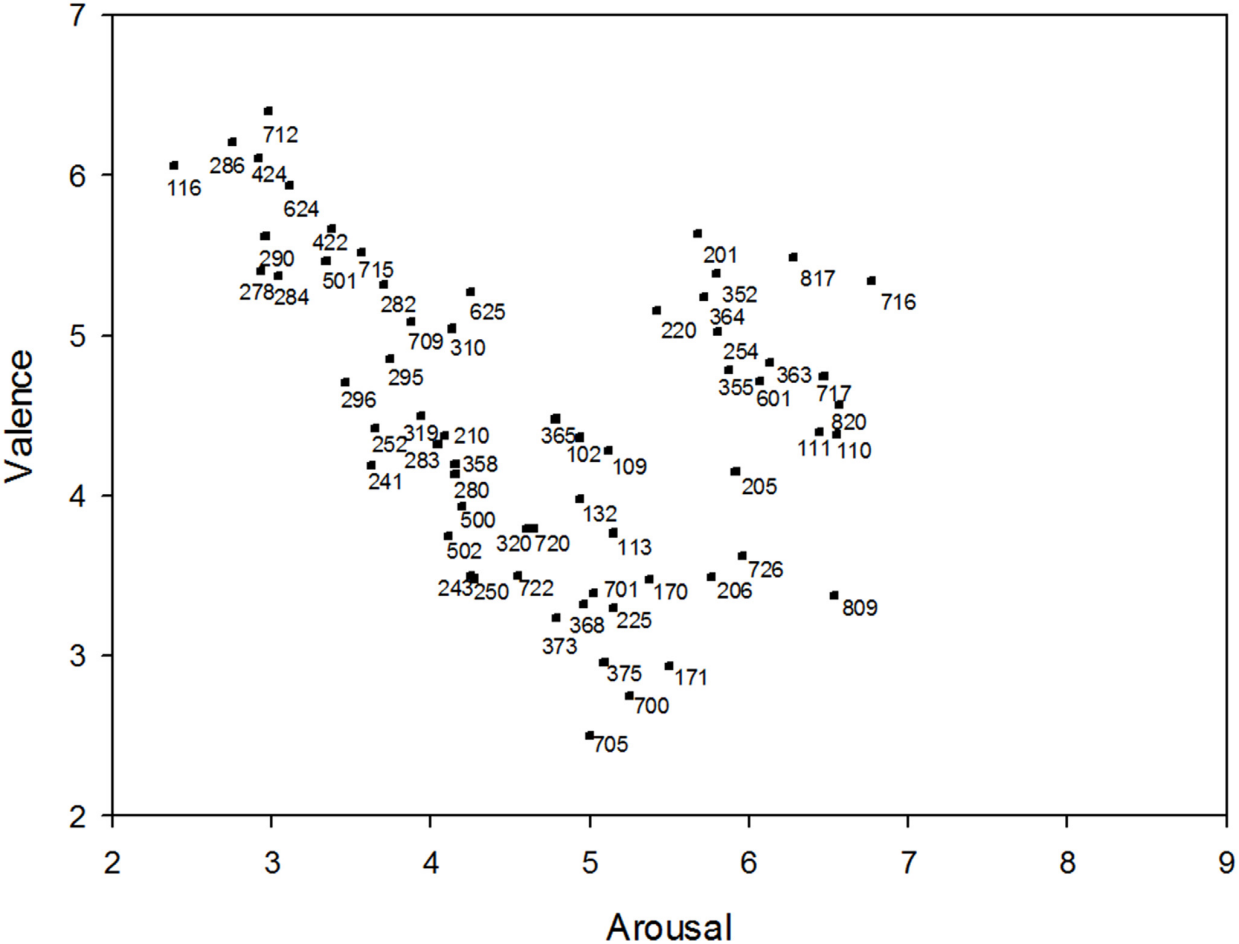
The distribution of the mean valence and arousal ratings elicited by IADS-2 sounds during the experiment. The numbers showed in the figure are the sound ids of the used sounds (these sounds can be found in the IADS-2 database [42] using the sound ids).

At the end of the training (i.e. the last typed sequence), the keystroke duration was significantly shorter (107.05 ms ± 22.56), t(40) = 6.31, p < .001 compared to the first typed sequence (115.18 ms ± 20.21). Moreover, the keystroke latency was significantly shorter (125.21 ms ± 43.39), t(40) = 2.31, p < .05 compared to the first typed sequence (215.64 ms ± 106.84). In Fig 2, each of the IADS sound was plotted in terms of its mean valence and arousal rating obtained from all the subjects. It is clear that the utilized sounds evoked reactions across a wide range of each dimension. The U-shaped relation between the valence and arousal indicated that these IADS sounds elicited the subjects’ feelings of being annoyed or alarmed (i.e. reporting negative valence with medium arousal), but not being angry (i.e. reporting negative valence with high arousal) and not being tired, sad, or bored (i.e. reporting negative valence with low arousal). The mapping of the valence-arousal space to possible discrete emotional states was derived from previous studies [53, 54] (interested readers are recommended to [55] for latest experimental results).

The descriptive statistics of the influence of emotion on keystroke duration are provided in Table 1. The keystroke duration data was submitted to a two-way Repeat Measures ANOVA. The ANOVA results are provided in Part A of Table 2. Statistically significant difference was found in the main effect Arousal. These results support the hypothesis that keystroke duration is influenced by emotional states. The percentage of the variability in the keystroke duration associated with the Arousal (η^2^) is 9.14 (after removing the effects of individual differences). The keystroke duration was significantly longer, t(40) = 2.30, p < .0135 when arousal was rated as low (108.76 ms ± 24.52) compared to when arousal was rated as high (106.70 ms ± 23.80). The ANOVA result of the dataset that includes the excluded 11 subjects by imputing all the missing values by using average values are also presented in Part B of Table 2.

**Table 1:** Descriptive statistics of keystroke duration under independent variables Valence x Arousal.

Valence	Arousal	Mean	Std. Error	95% Confidence Interval	
				Lower Bound	Upper Bound
negative	low	0.1141	0.0006	0.1130	0.1153
	medium	0.1138	0.0008	0.1121	0.1155
	high	0.1076	0.0005	0.1066	0.1086
neutral	low	0.1101	0.0005	0.1092	0.1111
	Medium	0.1134	0.0014	0.1106	0.1161
	High	0.1078	0.0012	0.1053	0.1102
positive	Low	0.1095	0.0006	0.1083	0.1106
	Medium	0.1071	0.0010	0.1052	0.1091
	High	0.1077	0.0007	0.1063	0.1091

**Table 2:** Repeated measures 3 (Valence: negative, neutral, positive) x 3 (Arousal: low, medium, high) ANOVA table for keystroke duration.

Source of Variance	SS	df	MS	F	P
Part A. (The ANOVA result of the dataset that excludes 11 subjects which contains over 3 empty cells)					
Subjects	0.208	40	0.005		
Valence	(SS < 0.001)	2	(MS < 0.001)	0.389	0.6792
Error(Valence)	0.003	80	(MS < 0.001)		
Arousal*	(SS < 0.001)	2	(MS < 0.001)	4.025	0.0216
Error(Arousal)	0.003	80	(MS < 0.001)		
Valence x Arousal	(SS < 0.001)	4	(MS < 0.001)	0.492	0.7413
Error(Valence x Arousal)	0.006	160	(MS < 0.001)		
Total	0.220	368			
Part B. (The ANOVA result of the dataset that contains all subjects)					
Subjects	0.264	51	0.005		
Valence	(SS < 0.001)	2	(MS < 0.001)	1.527	0.222
Error(Valence)	0.004	102	(MS < 0.001)		
Arousal**	(SS < 0.001)	2	(MS < 0.001)	4.845	0.0098
Error(Arousal)	0.003	102	(MS < 0.001)		
Valence x Arousal	(SS < 0.001)	4	(MS < 0.001)	1.267	0.2843
Error(Valence x Arousal)	0.006	203	(MS < 0.001)		
Total	0.279	466			

* p < .05

** p < .01

The descriptive statistics of the influence of emotion on keystroke latency are provided in Table 3. This keystroke latency data was submitted to a two-way Repeat Measures ANOVA. The ANOVA results are provided in Part A of Table 4. Statistically significant difference was also found in the main effect Arousal, but not the Valence and Valence by Arousal interaction. These results support the hypothesis that keystroke latency is influenced by emotional states, specifically, influenced by the arousal. The percentage of the variability in the keystroke latency associated with the Arousal (η^2^) is 11.48 (after removing the effects of individual differences). The keystroke latency was significantly longer when arousal was rated as medium (107.98 ± 38.44) compared to both when arousal was rated as low (103.26 ms ± 37.64; t(40) = 2.91, p < .0029) and when arousal was rated as high (104.34 ms ± 39.30; t(40) = 2.37, p < .0115). The ANOVA result of the dataset that includes the excluded 11 subjects by imputing all the missing values by using average values are also presented in Part B of Table 4.

**Table 3:** Descriptive statistics of keystroke latency under independent variables Valence x Arousal.

Valence	Arousal	Mean	Std. Error	95% Confidence Interval	
				Lower Bound	Upper Bound
negative	Low	0.1025	0.0013	0.1000	0.1050
	Medium	0.0968	0.0020	0.0927	0.1008
	High	0.1084	0.0012	0.1060	0.1108
neutral	Low	0.1055	0.0010	0.1034	0.1076
	Medium	0.0990	0.0028	0.0933	0.1046
	High	0.0995	0.0025	0.0944	0.1046
positive	Low	0.1071	0.0014	0.1044	0.1099
	Medium	0.1077	0.0021	0.1035	0.1118
	High	0.1032	0.0014	0.1003	0.1060

**Table 4:** Repeated measures 3 (Valence: negative, neutral, positive) x 3 (Arousal: low, medium, high) ANOVA table for keystroke latency.

Source of Variance	SS	df	MS	F	P
Part A. (The ANOVA result of the dataset that excludes 11 subjects which contains over 3 empty cells)					
Subjects	0.507	40	0.013		
Valence	(SS < 0.001)	2	(MS < 0.001)	0.212	0.809
Error(Valence)	0.011	80	(MS < 0.001)		
Arousal**	0.001	2	0.001	5.187	0.0076
Error(Arousal)	0.011	80	(MS < 0.001)		
Valence x Arousal	(SS < 0.001)	4	(MS < 0.001)	0.592	0.6691
Error(Valence x Arousal)	0.032	160	(MS < 0.001)		
Total	0.564	368			
Part B. (The ANOVA result of the dataset that contains all subjects)					
Subjects	0.620	51	0.012		
Valence	(SS < 0.001)	2	(MS < 0.001)	0.431	0.6512
Error(Valence)	0.021	102	(MS < 0.001)		
Arousal	(SS < 0.001)	2	(MS < 0.001)	0.969	0.3828
Error(Arousal)	0.016	102	(MS < 0.001)		
Valence x Arousal	(SS < 0.001)	4	(MS < 0.001)	0.765	0.5490
Error(Valence x Arousal)	0.035	203	(MS < 0.001)		
Total	0.693	466			

** p < .01

The descriptive statistics of the influence of emotion on accuracy data (0 for incorrectly typed sequence and 1 for correctly typed sequence) of all sequences typed are provided in Table 5. This accuracy rate data was submitted to a two-way Repeat Measures ANOVA. The ANOVA results are provided in Part A of Table 6. Although the p-values are small (i.e. 0.1 and 0.2), no statistically significant difference was found. This result rejects the hypothesis that the accuracy rate of keyboard typing is influenced by emotional states. The ANOVA result of the dataset that includes the excludes 11 subjects by imputing all the missing values by using average values are also presented in Part B of Table 6. Notably, the variance contributed by valence and arousal for keystroke duration, keystroke latency, and accuracy rate are all small (see Tables 2, 4, and 6), compared to the individual variability.

**Table 5:** Descriptive statistics of accuracy rate under independent variables Valence x Arousal

Valence	Arousal	Mean	Std. Error	95% Confidence Interval	
				Lower Bound	Upper Bound
negative	low	0.9308	0.0111	0.9085	0.9531
	medium	0.9106	0.0182	0.8741	0.9470
	high	0.9029	0.0123	0.8783	0.9276
neutral	low	0.9440	0.0088	0.9263	0.9616
	medium	0.9495	0.0221	0.9053	0.9937
	high	0.9423	0.0230	0.8964	0.9883
positive	low	0.9482	0.0105	0.9271	0.9693
	medium	0.9235	0.0204	0.8826	0.9644
	high	0.9003	0.0167	0.8668	0.9338

**Table 6:** Repeated measures 3 (Valence: negative, neutral, positive) x 3 (Arousal: low, medium, high) ANOVA table for accuracy rate of keyboard typing

Source of Variance	SS	df	MS	F	P
Part A. (The ANOVA result of the dataset that excludes 11 subjects which contains over 3 empty cells)					
Subjects	2.982	40	0.075		
Valence	0.048	2	0.024	2.343	0.1026
Error(Valence)	0.820	80	0.010		
Arousal	0.028	2	0.014	1.642	0.2001
Error(Arousal)	0.685	80	0.009		
Valence x Arousal	0.027	4	0.007	0.715	0.583
Error(Valence x Arousal)	1.535	160	0.010		
Total	6.125	368			
Part B. (The ANOVA result of the dataset that contains all subjects)					
Subjects	3.486	51	0.068		
Valence	0.061	2	0.031	1.877	0.1583
Error(Valence)	1.662	102	0.016		
Arousal	0.029	2	0.015	1.517	0.2243
Error(Arousal)	0.981	102	0.010		
Valence x Arousal	0.041	4	0.010	1.064	0.3753
Error(Valence x Arousal)	1.970	203	0.010		
Total	8.230	466			

## Discussions

Previous studies [1, 37, 40, 41] have highlighted the possibility of using keyboard typing data to detect emotions. Specifically, keystroke duration, keystroke latency, and accuracy rate of keyboard typing were used as input features for model building. These results have led to three hypothesized relationships. That are, the relationship between keystroke duration and emotion, the relationship between keystroke latency and emotion, and the relationship between accuracy rate of keyboard typing and emotion. Hence, the current study tests these three hypothesized relationships. The results of our experiment using the fix target typing text and the 63 stimuli selected from the IADS-2 database [42] supports the hypotheses that the keystroke duration and latency are influenced by arousal. Our finding supports previous studies [1, 37, 40, 41] that aimed to build classification model of emotions through keystroke data. Shorter keystroke duration is found when arousal is high (106.70 ms ± 23.80) compared to the keystroke duration when arousal is low (108.76 ms ± 24.52), which implies that button presses may have been carried out with less strength [21] when arousal was low. This result indicates an increased keystroke duration when the subjects experienced tired, sad, or bored [53, 54]. The result is in line with the findings reported by [21, 38], which suggest a longer keystroke duration accompanied with negative emotional state. In addition, we found a slowest keystroke latency (i.e. keyboard typing speed) when arousal is medium. This finding may suggest that negative emotions lead to a slower keyboard typing speed, since the result in Fig 2 implies that the subjects may have more opportunity of experiencing negative valence when arousal rated as medium during the experiment. The result of recent study [56] that observed the changes in keyboard typing speed due to emotion, corroborates this finding. The current study further extends the results obtained in [44] which demonstrated the effect of visual stimuli induced arousal on keystroke duration and latency, by showing that the effect of auditory stimuli induced arousal on keystroke duration and latency. This shows that the effect of emotion on keystroke duration and latency appears for both the emotion induced by visual stimuli and the emotion induced by auditory stimuli, which were believed to be interpreted by human brain through different biological pathways [57].

**Fig 3:**
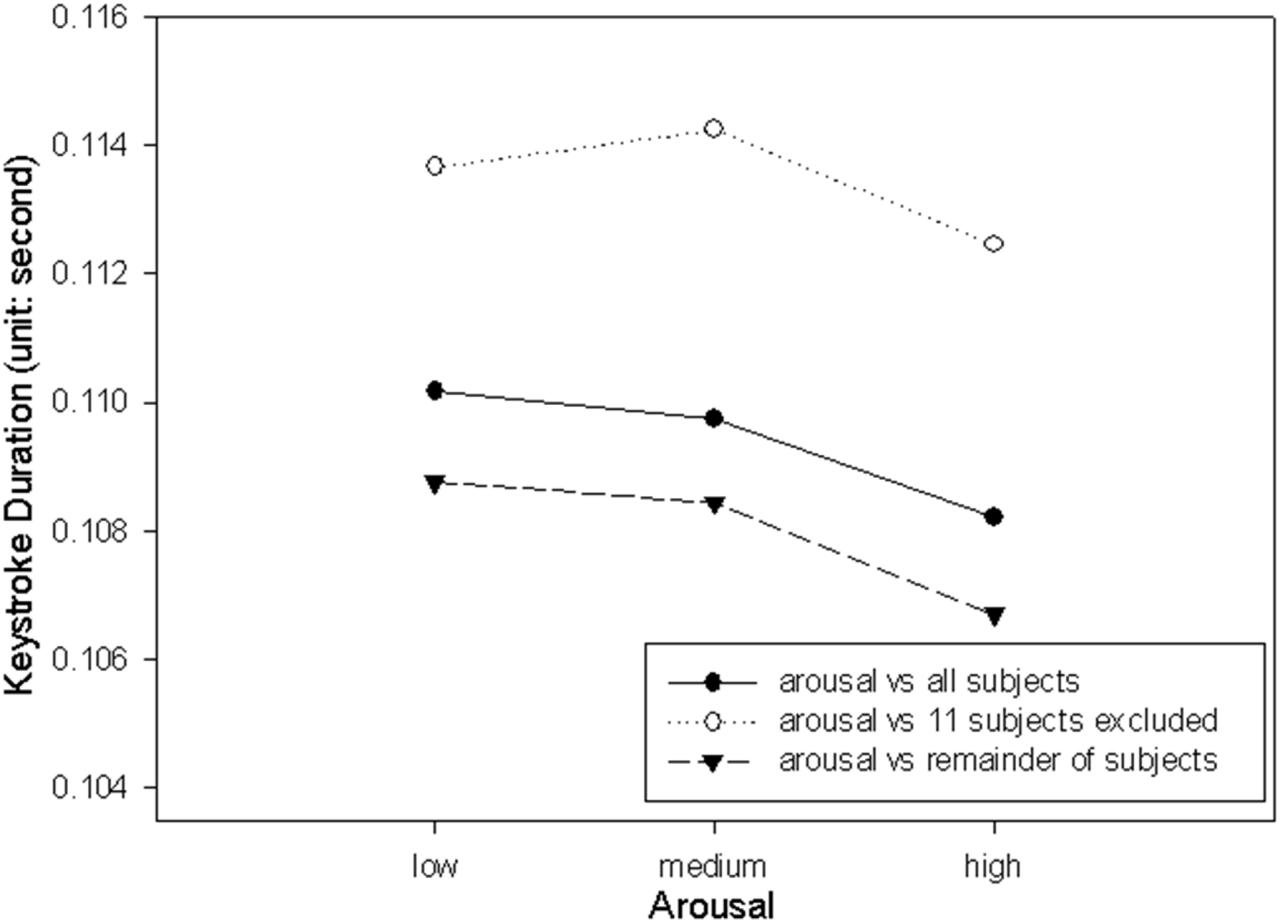
Keystroke duration with respect to arousal.

**Fig 4:**
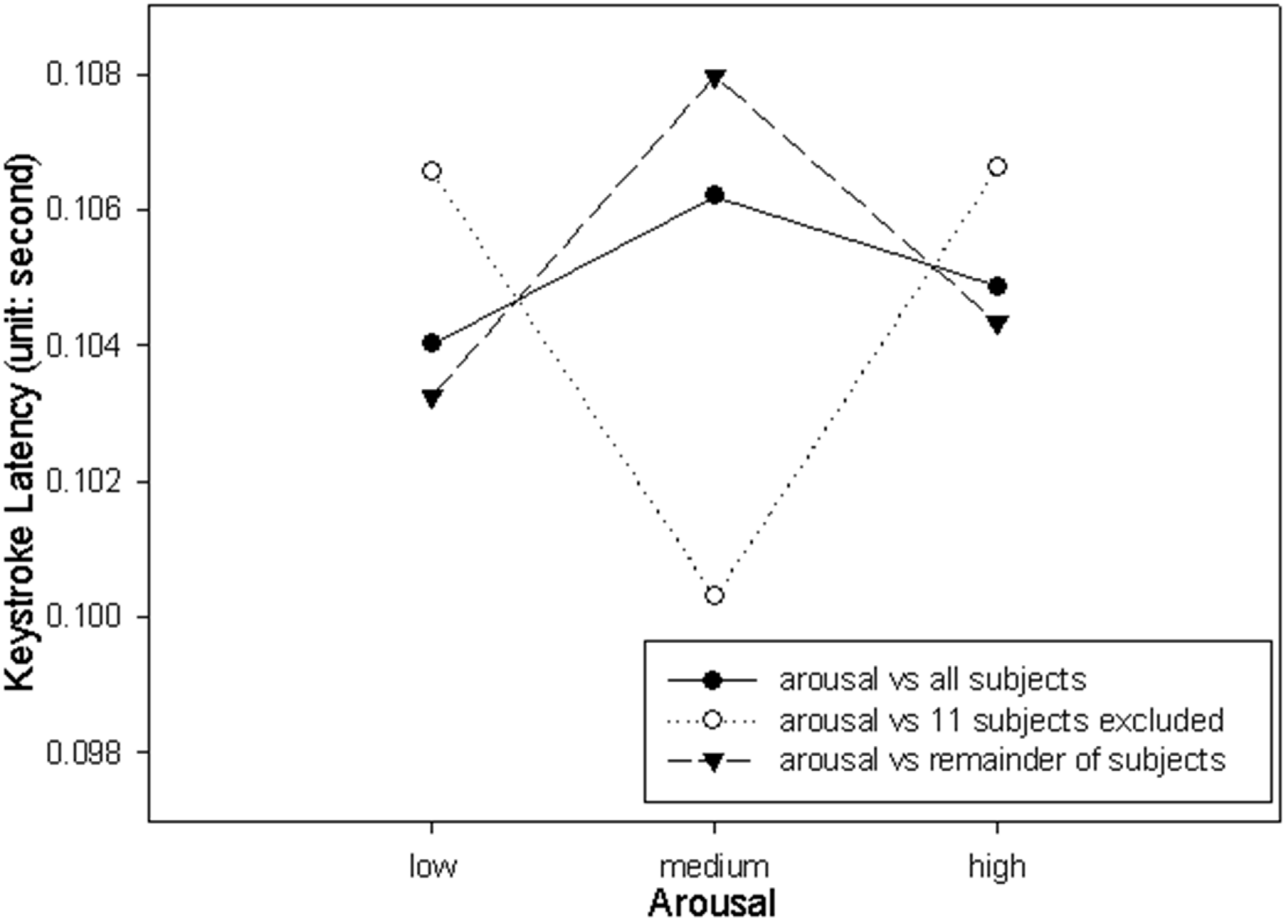
Keystroke latency with respect to arousal.

The results of the current study may be critical while they were obtained from the analysis that with eleven subjects excluded, despite the fact that these subjects contain 47.48% (i.e. (6 + 6 + 6 + 5 + 4 + 4 + 4 + 3 + 3 + 3 + 3) ∕ (11 * 9)) missing values in their data, and to include these subjects in an analysis by imputing numerous missing values should lead to unreliable results in regard to the research objectives (i.e. to examine the keystroke dynamics in the 3 x 3 emotional conditions) of the current study. The reason of the result for being critical is that the exclusion of eleven subjects may increase the likelihood of detecting the desired effects. Hence, although for auditory stimuli we found that the main effect of arousal exceeds significant level for both keystroke duration and latency, the readers should generate their own view of the significance of these results. It is worth to note that while arousal was significant in both analyses that with and without those 11 subjects, the arousal was not significant for keystroke latency when the 11 subjects were included in the analysis. Figs 3 and 4 shows the plotting of the arousal data against keystroke duration and latency, respectively, with the data points of the excluded 11 subjects marked. The plotting in Fig 3 indicates that the pattern shown by the 11 subjects excluded from the analysis is similar to the pattern shown by the remainder of the subjects. On the other hand, the plotting in Fig 4 indicates that the pattern of the 11 subjects excluded from the analysis is opposite to the remainder of subjects. The finding suggests that the 11 subjects excluded from the analysis may have acted in patterns with respect to arousal different from the remainder subjects, and this should be the cause of the main effect Arousal for not being significant for keystroke latency. The different patterns could be caused by individual difference. Another possible explanation to the different patterns is that the subjects whose emotion was hard to be elicited, may have physiological patterns with respect to their emotional state different from the physiological patterns of normal subjects [58].

The small variance contributed by valence and arousal (see MSs of valence and arousal in Tables 2, 4, and 6) compared to the variance contributed by individual difference (see MSs of subjects in Tables 2, 4, and 6), suggests that although previous studies [1, 37, 40, 41] used to build intelligent systems that act user-independently in detecting emotional states of users based on the keystroke dynamics, the accuracy rate of the detection could be further improved if personalized models (i.e. taking user id as an input attribute/explanatory variable for model building, or simply build classification models for each user instead of one model for all people) [59] are utilized. The observations of large variance contributed by individual difference is in line with previous findings [38] in regard to the effect of facial feedback induced emotions on keystroke duration and latency, which suggested that the patterns of the effect of emotion on each subject were different.

To summarize, the research question about the three hypothesized relationships between emotions and keystroke dynamics are answered by using traditional statistical methods instead of using ML algorithms. The evidence found in the current study supports the hypotheses that keystroke duration and latency are influenced by arousal, whereas failed to prove the hypothesized relationship between accuracy rate of keyboard typing and emotions (despite the fact that the p-values for valence and arousal are both small). The findings of the current study are expected to support the development in technology that detects users’ emotion through keystroke dynamics, which may be applied to various applications in HCI in the near future.
